# Evaluation of the Effectiveness of Virtual Telephone Consultations Against Traditional Face-to-Face Consultations in Spine Surgery Using an Objective Metric

**DOI:** 10.7759/cureus.69433

**Published:** 2024-09-15

**Authors:** Siddhartha Murhekar, Sanjana Relwani, Steve Lau, Siddharth Virani

**Affiliations:** 1 Trauma and Orthopaedics, East Kent Hospitals University NHS Foundation Trust, Canterbury, GBR; 2 Trauma and Orthopaedics, Barts Health NHS Trust, London, GBR

**Keywords:** ashford clinic letter score, face to face consultation, inter-observer reliability, spine clinic, telephone medicine

## Abstract

Introduction: Coronavirus disease 2019 (COVID-19) proved to be a catalyst in the paradigm change from face-to-face (F2F) to virtual consultations in trauma and orthopaedics. This study evaluates the efficacy of telephone consultations versus F2F reviews in an elective spine clinic.

Methods: In this retrospective study, the clinic letters from elective spinal clinics were conducted over one month. Patients with lumbar spine pathology were included, divided into telephone and F2F groups, and further categorized into new referrals and follow-ups. The Ashford Clinic Letter Score (ACLS) was used to assess the efficacy of consultations.

Results: Out of 126 spinal patients reviewed, 92 met the inclusion criteria. Of these, 47 were F2F and 45 were telephone consultations. The mean satisfaction scores for all patients were 7.60 for F2F and 7.42 for telephone consultations, with no significant difference (p=0.202). New patient satisfaction scores were 7.64 for F2F and 7.35 for telephone (p=0.284), and follow-up scores were 7.58 for F2F and 7.48 for telephone (p=0.530). The results showed that there was no difference between face-to-face and virtual consultations.

Conclusion: Telephone consultations are nearly as effective as F2F consultations in elective spine clinics, particularly for follow-up patients. The findings support the viability of telemedicine as an economical and efficient alternative method instead of traditional F2F consultations in orthopaedic practice. Further research is needed to explore patient satisfaction and outcomes in other spinal pathologies and to optimise patient stratification for telemedicine.

## Introduction

The outpatient clinical practice in trauma and orthopaedics has undergone significant changes accelerated by the COVID-19 pandemic [1]. Telemedicine consultations have increased considerably, with an overwhelming shift from in-person clinics to virtual appointments implemented among trauma and orthopaedic centres across the United Kingdom [2]. Among spine patients specifically, the coronavirus disease 2019 (COVID-19) pandemic has been a major catalyst in the shift to remote consultations; in 2020, over 30% of spine surgeons globally carried out more than half of their clinical consultations virtually [3]. Telemedicine consultations with spine and orthopaedic patients can be particularly valuable because they are more convenient and accessible than face-to-face (F2F) consultations, removing geographic barriers to care and allowing patients to receive a professional diagnosis and treatment recommendation from the comfort of their homes. Despite this, physical examinations have long been regarded as crucial in diagnosing and treating musculoskeletal disorders, and the rapid shift in practice patterns towards virtual consultation has posed a unique challenge for spine surgeons [4]. Trauma and orthopaedic surgery has been at the forefront of accepting telehealth such as telephonic appointments, virtual fracture clinics, and video consultations [5]. There are a limited number of studies that have objectively compared the efficacy of virtual reviews against face-to-face reviews. Further, this number is even more limited in the context of spine surgery. Taking this forward, the primary aim of the research is to evaluate the efficacy of telephone medicine consultations against actual clinical reviews using objective metrics.

The tool used to achieve this goal is the Ashford Clinic Letter Score (ACLS) [6]. This score is a valid, reliable, and reproducible metric that assesses the efficacy of a consultation in trauma and orthopaedics. It does so using the clinic letter or clinic notes made by the surgeon. The ACLS is a standardised method of evaluating the quality of a consultation using clinic letters using parameters of evaluating symptoms to make a diagnosis, arranging pertinent investigations, and drawing up a treatment plan. This tool has been tested in elective knee and hip clinics and shoulder and elbow clinics. This concise tool is not only reliable but also easily reproducible, which helps in using it to assess the quality of consultations objectively. 

## Materials and methods

Elective spinal clinics conducted by a team consisting of a consultant, middle-grade trainees, and associate specialists over one month were assessed. Patients with pathology related to the lumbar spine were included. All other patients with cervical spondylosis, cervical myelopathy, spinal deformity, and cervical radiculopathy were not included in the clinic. All the patients were organised into two groups of telephone and face-to-face consultations, respectively. During the COVID-19 pandemic, we had shifted to full-time virtual clinics. As the pandemic evolved, the clinics were hybrid in nature with both face-to-face and telephonic consultations. Each group was further subdivided into new referrals for patients attending clinics for the first time and follow-up patients who were already known to the department.

Each consultation was scored appropriately using the clinic assessment tool, Ashford Clinic Letter Score [6]. This score is based on the clinic consultation letter/notes to determine its effectiveness. The score consists of four parameters, with each parameter being scored from 0 to 2. The first parameter is the ability to make a clear, working clinical diagnosis. The second parameter assesses if the relevant investigations needed are available. The third parameter analyses if an appropriate treatment plan could be formulated, while the final parameter judges the value of the consultation (Table 1). The mean of scores assessed by two independent assessors was considered for each component while formulating final scores (Table 2).

**Table 1:** The Ashford Clinic Letter Score (ACLS) *When a registrar/fellow reviews the patient Descriptors of each component of the Ashford Clinic Letter Score (maximum score of 2) used for scoring the clinical consultations. Virani S, Eastwood S, Holmes N, Shaeir M, Housden P. Objective assessment of the efficacy of telephone medicine consultations in dispensing elective orthopaedic care using a novel scoring tool. [6]

Component	Parameter	Component	Score
A	Making a diagnosis	Clear working clinical diagnosis	2
		Face-to-face review would improve the diagnosis	1
		No diagnosis	0
B	The consultation recognised	All relevant investigations are done and available	2
		Tests/Imaging done but need importing	1
		Clear which investigations are needed and ordered	1
		Not clear what is needed at this stage	0
C	Formulation of treatment plan	Treatment plan made	2
		A clear treatment plan requires face-to-face review	1
		Consultant input is needed within 3 weeks for a treatment plan*	1
		No treatment plan could be agreed	0
D	Consultations was valuable communication event	Yes	2
		No, but doctor-patient relationship is intact	1
		No and future treatment difficult due impairment of relationship	0
N/A	N/A	TOTAL Score (out of 8)	../8

**Table 2:** Guidelines on allotting scores to the Ashford Clinic Letters Score *Although the score here is 2, the score in component B would be 1 MRI- Magnetic Resonance Imaging, CT scan - Computed tomography scan Virani S, Eastwood S, Holmes N, Shaeir M, Housden P. Objective assessment of the efficacy of telephone medicine consultations in dispensing elective orthopaedic care using a novel scoring tool. [6]

Component	Parameter		Guidelines/Rules to allot scores	Scores
A	Making a diagnosis		Clear working diagnosis based on history and investigations available	2
			Improving post operatively	2
			Examination would help confirm the region of the pathology/inconclusive examination	1
			Post-operative patient worsening or not improving	1
			Cannot identify the region of pathology	0
B	Investigations		All needed investigations available	2
			Follow-up radiographs/bloods needed in few months	2
			Relevant investigations (MRI,CT,Bone Scan,etc) requested for	1
			Radiographs/bloods needed within 3 weeks	1
			Unsure what is needed	0
C	Treatment Plan		Listed for surgery	2
			Discharged	2
			Routine post-op follow-up arranged	2
			Follow-up to review pertinent investigations *	2
			Follow-up to see how patient is ‘getting along’	1
			A treatment plan cannot be agreed	0
D	Consultations was valuable communication event		Value of the consultation	
			Patient and doctor satisfied by the outcome	2
			Patient would have preferred face-to-face	1
			Surgeon/patient unhappy/dissatisfied	1
			The consultation made further treatment more difficult due to the relationship breakdown	0

The scores for each parameter and the total scores were recorded in Microsoft Excel (Redmond, WA, USA). The mean scores and standard deviations were calculated. While using an unpaired t-test, the confidence interval was found to be 95%, assuming statistical significance. The statistical difference between the total score for consultations conducted face-to-face and by telephone was assessed. The difference was also assessed between the two modalities of consultation for sub-groups of new referrals and follow-up patients.

## Results

Of the 126 spinal patients reviewed in the given period, 92 met the inclusion criteria. The most common clinical diagnosis was sciatica associated with disc disease. Other common patients included those with lumbar canal stenosis, facetal back pain, postoperative patients, and degenerative lumbar spine disease.

**Figure 1:**
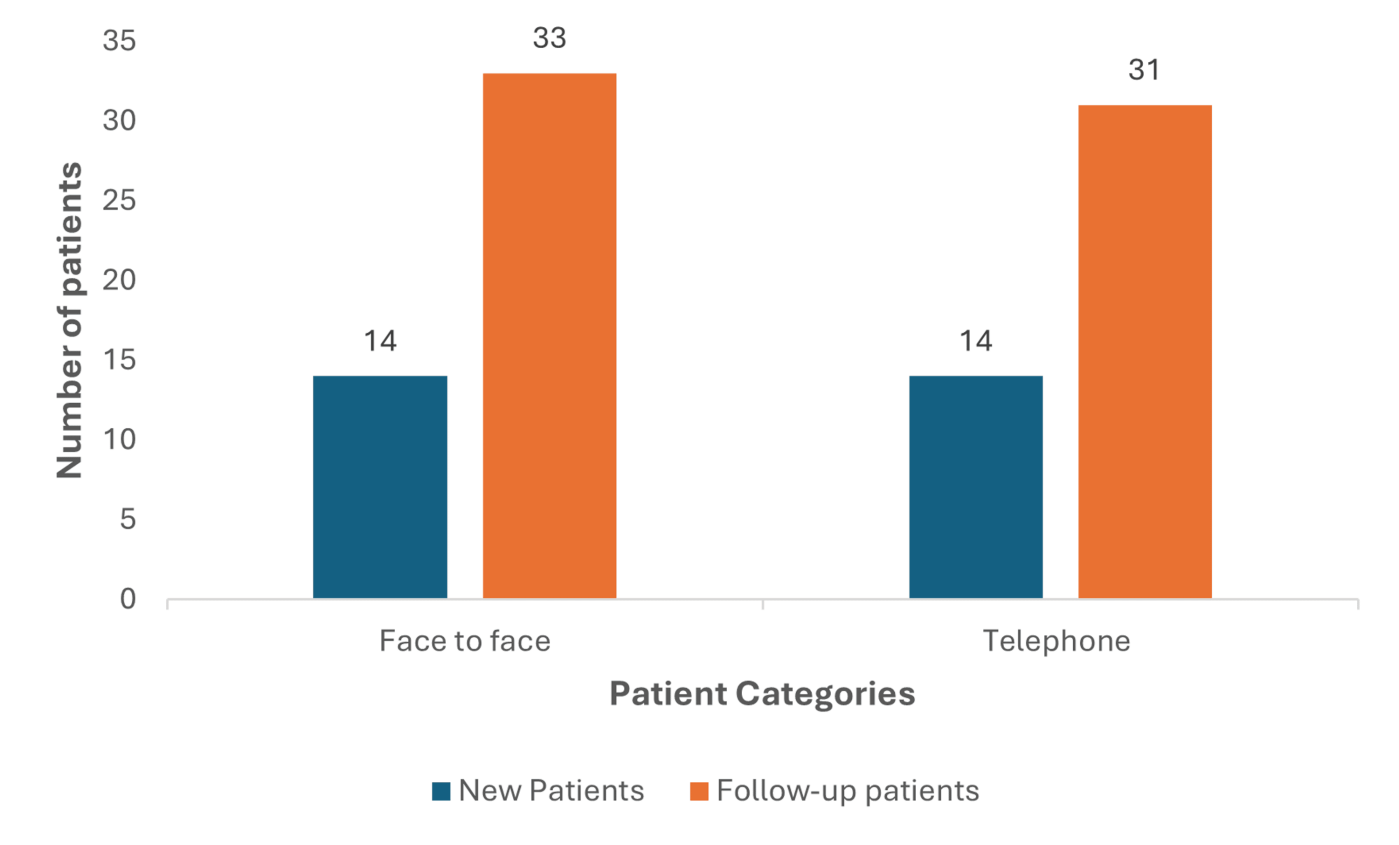
Patient distribution Distribution of new and follow-up patients with elective lumbar spine pathologies in the face to face and telephone consultation groups

Of the total consultations, 47 were face-to-face while 45 consultations were by telephone. Among the in-person consultations, 14 were new patients and 33 were follow-up patients; among the 45 telephonic consultations, there were 14 new patients and 31 were follow-up patients (Figure 1).

**Figure 2:**
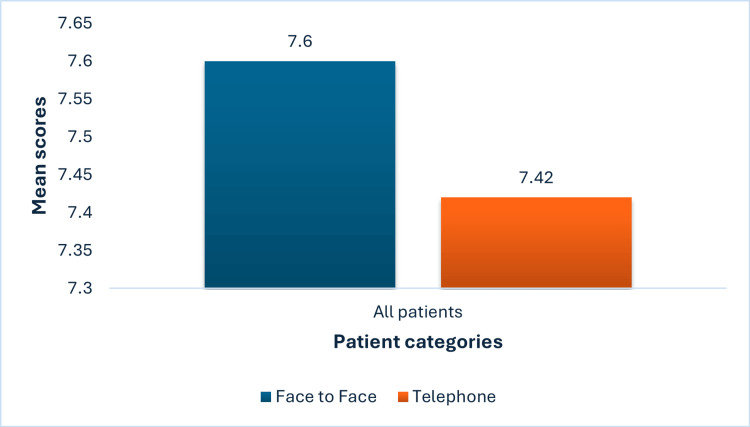
Mean total score of face-to-face and telephone clinics The mean Ashford Clinic Letter Score (ACLS) (maximum score of 8) was used as a measure of the efficacy of a clinic consultation in face-to-face and telephone consultations suggesting a difference that is not statistically significant (p=0.202)

The study compared patient satisfaction scores between face-to-face and telephone consultations across three groups: all patients, new patients, and follow-up patients. An ANOVA test was performed assuming 95% confidence intervals between these groups. For all patients, the mean satisfaction score was 7.60 (out of a maximum score of 8) for face-to-face consultations and 7.42 for telephone consultations, with no statistically significant difference (p = 0.202) (Figure 2).

**Figure 3:**
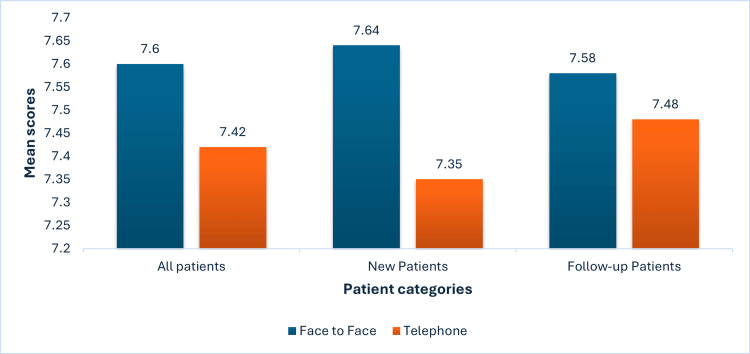
Mean scores for new and follow-up patients The mean Ashford Clinic Letter Score (ACLS) (maximum score of 8) used as a measure of efficacy of a clinic consultation in face-to-face and telephone consultations suggesting a difference that is not statistically significant in both new (p=0.284) and follow-up consultations (p=0.530)

New patients' mean satisfaction scores for face-to-face and telephone were 7.64 and 7.35, respectively, and were also not significantly different (p = 0.284). Follow-up patients' mean satisfaction scores were 7.58 for face-to-face and 7.48 for telephone, with a non-significant p-value of 0.530 (Figure 3).

Standard deviations and variances were slightly higher for telephone consultations across all groups, indicating more variability in those scores. Overall, no significant differences were found between face-to-face and telephone consultations. This shows that there was no significant difference between face-to-face and telephonic consultations in the spinal surgery clinics (Table 3).

**Table 3:** The p-values detailing standard deviation and variance between face-to-face and telephone clinics

		Face to Face	Telephone	p-value
All patients	Mean	7.60	7.42	p=0.202
	S.D.	0.56	0.77	
	Variance	0.32	0.59	
New Patients	Mean	7.64	7.35	p=0.284
	S.D.	0.47	0.81	
	Variance	0.22	0.65	
Follow-up Patients	Mean	7.58	7.48	p=0.530
	S.D.	0.59	0.72	
	Variance	0.35	0.52	

On a detailed analysis of the scores, telephonic consultations were comparable to face-to-face consultations when new plans were drawn up in patients who came to the clinic for the first time (score 1.85 out of maximum score 2). Remarkably, the value of consultation had no significant difference in telephonic and face-to-face consultation, which is scored by patient and surgeon satisfaction from the clinic.

**Figure 4:**
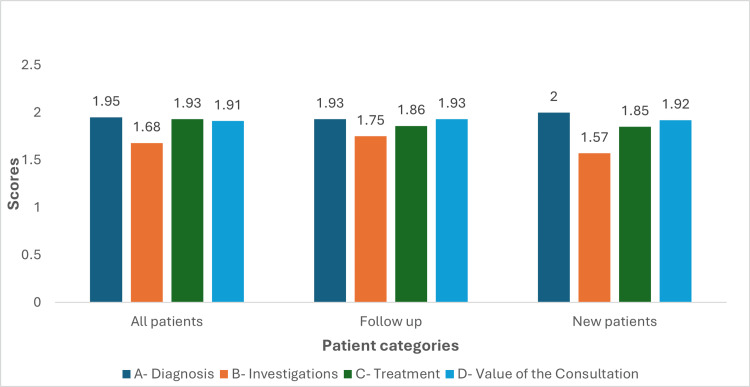
Performance of clinical modalities in face-to-face consultations Performance of each component of the Ashford Clinic Letter Score (ACLS) (Diagnosis, Investigations, Treatment plan and Value of consultations) in new and follow-up face-to-face consultations.

**Figure 5:**
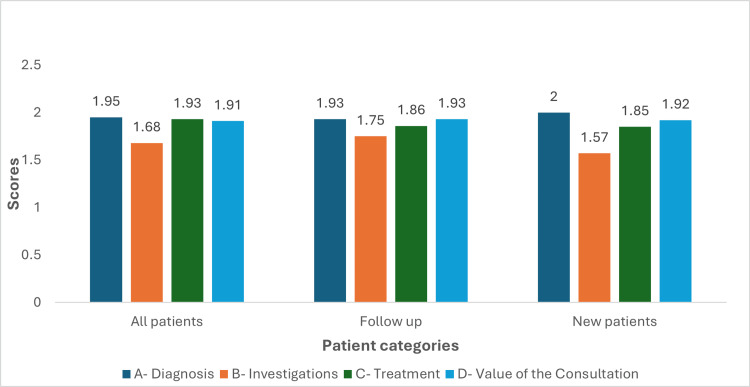
Performance of clinical modalities in telephonic consultations Performance of each component of the Ashford Clinic Letter Score (ACLS) (Diagnosis, Investigations, Treatment plan and Value of consultations) in new and follow-up telephone consultations.

The outcome scores from the individual assessment using the ACLS tool were comparable between face-to-face and telephonic consultations, wherein both of them contributed to providing specialised care for patients (Figures 4, 5).

## Discussion

From the start of the COVID-19 pandemic, there was a significant impact on healthcare delivery systems. As the number of hospital admissions increased, these systems began to struggle [7]. However, the pandemic also catalysed the increased use of telehealth services to meet the growing demand [8]. Virtual clinics provided solutions to many challenges patients faced during this difficult period. As the pandemic subsided, the advantages and broad applicability of telehealth were recognised across various specialities, including surgical fields such as orthopaedics [5]. Multiple studies and systematic reviews have concluded that high-quality care can be delivered via telemedicine while maintaining good clinical outcomes. Additionally, telemedicine has proven to be cost-effective, reducing the travel burden on patients and maintaining high levels of patient satisfaction [9,10]. There was no change in the proportion of no-show visits pre- and during the COVID-19 pandemic [11].

There is ample evidence in the literature supporting the use of telephone medicine to improve patient satisfaction with good clinical outcomes [9]. Tools such as Sheffield Assessment Instrument Letters (SAIL) and the ACLS were widely used to assess the effectiveness of clinic consultations. The SAIL was developed primarily for paediatrics using a 20-point questionnaire found to have wider applications. On the contrary, the ACLS tool is a score subdivided into four components of consultations by scoring the letters written to the General Practitioner (GP) [12,13]. The maximum score allocated to each component is 2, and hence the total maximum score is 8. The ‘diagnosis’ component essentially means a working clinical diagnosis, while the ‘treatment plan’ explains what was expected in the next subsequent consultations of the patient, not necessarily being definitive management. The paper also described guidelines for scoring each of the four components and the score pertinent to each section [6]. 

The ACLS tool has been successfully used to assess the efficacy of consultations for elective hips and knee clinics, shoulder and elbow clinics, and general trauma clinics. The outcomes of these studies emphasised that the use of telemedicine is cost-effective and patient stratification remains key to the success of telephone consultations [14,15]. 

Regarding patients with lumbar spine pathologies, we noted that the patients with face-to-face clinical reviews consistently performed better than telephonic reviews. However, this difference was not statistically significant. We also noted more variability and a wider distribution of scores with telephonic consultations which may suggest there is a cohort that could do better with face-to-face consultations. Sathiyakumar et al. in their prospective randomized controlled trial found out that after six-week and six-month follow-up appointments, there was no difference in patient satisfaction between telemedicine and in-person clinics [16].

Sibanda et al. assessed the effectiveness of video and telephonic consultations in upper limb clinics (shoulder and elbow) and found that both were as effective as face-to-face consultations. They used the ACLS system to make these comparisons, which included new as well as follow-up patients and found the tool to be reliable [13]. Harno et al., in their prospective comparative study of 419 patients in primary and secondary care centres, found that telemedicine was not only cost-effective but also clinically effective for orthopaedic patients [17].

The Lightsey et al. study was based on whether the surgical planning changed between virtual clinics and face-to-face consultations. They found out that out of 33 patients included in the study, there was no change in surgical plan in 31 patients (94%) [4]. There were similar studies that concluded that telemedicine evaluations can be used in the stage of preoperative assessment of spine patients and to sketch future surgical plans [18-20]. Haider et al. did an in-depth systematic review to assess the impact of telemedicine in trauma and orthopaedic surgery during the pandemic and its future applications. They concluded that telemedicine, being safe and cost-effective, could be used in the clinical assessment of patients with spinal pathologies while maintaining high patient satisfaction [2,21]. Implenting digital health, especially for follow-up patients was concluded to be cost-effective with no negative patient impact as described in the randomized trial by Muschol et al. [22].

Our paper is the first paper to assess the efficacy of telephonic versus face-to-face spinal surgery clinics. To avoid inter-observer variability, the clinics were run by the same team throughout rather than multiple teams. We used letters from a single consultant clinic ordained by consultants, middle-grade staff, and associate specialists. To enhance the generalizability of the results, future studies could include multiple consultants and teams. As this study was carried out retrospectively, there can be weakness in the data collection as against a blinded randomised prospective trial. The study only included patients with lumbar pathology and this can limit the generalisability of data to all spinal clinics which as multi-level pathology of the spine. There is also a selection bias towards less complex, single-level pathologies while more complex cases are generally seen in face-to-face clinics. Future research should evaluate the incidences of missed complications along with other disadvantages of telephonic consultations. There is also a need to prospectively identify patients who are more likely to miss or fail to follow up on telephone consultations compared to face-to-face consultations. Patient surveys could be a valuable tool to determine the missing pieces of the puzzle and gain insights into patient's perspectives of telemedicine. There is a need to evaluate the efficacy and patient satisfaction of video and telephonic consultations and to compare them with face-to-face consultations.

We used a consultation letter from the clinic as a proxy for the consultation. Thus, the outcome of the ACLS score may vary depending on multiple factors, such as the availability of clinic time. We used letters from single consultant clinics run by consultations, middle-grade trainees, and associate specialists. The biggest obstacle in setting up telemedicine is the inertia towards a shift from face-to-face clinics. There is a need for further studies including a larger sample size for expanding it to the general population. However, the potential for cost-effectiveness, including a significant reduction in loss to follow-up patients, makes it a compelling option.

## Conclusions

The key conclusions of the study are that remote telephone consultations perform as well as face-to-face consultations with regards to elective spine clinics focusing on lumbar spine pathologies. These findings are applicable to both follow-up and new patients although the difference is wider with newer patients. We would like to suggest that in today’s day and age where remote and virtual consultations are being used more frequently, this study objectively validates the efficacy of telephonic consultations in elective spine clinics. Further multi-centre studies assessing practice across a wider cohort of consultants would be needed to draw firm conclusions. The utility of the Ashford Clinic Letter Score could be extended for this purpose.

## References

[1] Rhind JH, Ramhamadany E, Collins R, Govilkar S, Dass D, Hay S (2020). An analysis of virtual fracture clinics in orthopaedic trauma in the UK during the coronavirus crisis. EFORT Open Rev.

[2] Haider Z, Aweid B, Subramanian P, Iranpour F (2022). Telemedicine in orthopaedics during COVID-19 and beyond: a systematic review. J Telemed Telecare.

[3] Louie PK, Harada GK, McCarthy MH (2020). The impact of COVID-19 pandemic on spine surgeons worldwide. Global Spine J.

[4] Lightsey HM 4th, Crawford AM, Xiong GX, Schoenfeld AJ, Simpson AK (2021). Surgical plans generated from telemedicine visits are rarely changed after in-person evaluation in spine patients. Spine J.

[5] Makhni MC, Riew GJ, Sumathipala MG (2020). Telemedicine in orthopaedic surgery: challenges and opportunities. J Bone Joint Surg Am.

[6] Virani S, Eastwood S, Holmes N, Shaeir M, Housden P (2021). Objective assessment of the efficacy of telephone medicine consultations in dispensing elective orthopaedic care using a novel scoring tool. Surgeon.

[7] Pataki C, Singhal P, Dastidar AG (2021). UK-India telemedicine project and COVID-19. QJM.

[8] Mahtta D, Daher M, Lee MT, Sayani S, Shishehbor M, Virani SS (2021). Promise and perils of telehealth in the current era. Curr Cardiol Rep.

[9] Fahey E, Elsheikh MF, Davey MS, Rowan F, Cassidy JT, Cleary MS (2022). Telemedicine in orthopedic surgery: a systematic review of current evidence. Telemed J E Health.

[10] Tripod M, Tait M, Bracey J, Sexton K, Beck W, Wyrick TO (2020). The use of telemedicine decreases unnecessary hand trauma transfers. Hand (N Y).

[11] Siow MY, Walker JT, Britt E (2020). What was the change in telehealth usage and proportion of no-show visits for an orthopaedic trauma clinic during the COVID-19 pandemic?. Clin Orthop Relat Res.

[12] Crossley GM, Howe A, Newble D, Jolly B, Davies HA (2001). Sheffield Assessment Instrument for Letters (SAIL): performance assessment using outpatient letters. Med Educ.

[13] Fox AT, Palmer RD, Crossley JG, Sekaran D, Trewavas ES, Davies HA (2004). Improving the quality of outpatient clinic letters using the Sheffield Assessment Instrument for Letters (SAIL). Med Educ.

[14] Sibanda V, Onubogu I, Raad M, Virani S, Relwani J (2021). How effective are telephone and video consultations in shoulder and elbow clinics? Analysis using an objective scoring tool. Cureus.

[15] Raad M, Ndlovu S, Neen D (2021). Assessment of the efficacy of telephone medicine consultations in trauma and orthopaedics during COVID-19 using the Ashford Clinic Letter Score. Cureus.

[16] Sathiyakumar V, Apfeld JC, Obremskey WT, Thakore RV, Sethi MK (2015). Prospective randomized controlled trial using telemedicine for follow-ups in an orthopedic trauma population: a pilot study. J Orthop Trauma.

[17] Harno K, Arajärvi E, Paavola T, Carlson C, Viikinkoski P (2001). Clinical effectiveness and cost analysis of patient referral by videoconferencing in orthopaedics. J Telemed Telecare.

[18] Melnick K, Porche K, Sriram S (2023). Evaluation of patients referred to the spine clinic via telemedicine and the impact on diagnosis and surgical decision-making. J Neurosurg Spine.

[19] Ye IB, Thomson AE, Donahue J (2022). Similar accuracy of surgical plans after initial in-person and telemedicine evaluation of spine patients. World Neurosurg.

[20] Crawford AM, Lightsey HM, Xiong GX, Striano BM, Greene N, Schoenfeld AJ, Simpson AK (2021). Interventional procedure plans generated by telemedicine visits in spine patients are rarely changed after in-person evaluation. Reg Anesth Pain Med.

[21] Donnally CJ 3rd, Vaccaro AR, Schroeder GD, Divi SN (2021). Is evaluation with telemedicine sufficient before spine surgery?. Clin Spine Surg.

[22] Muschol J, Heinrich M, Heiss C (2023). Digitization of follow-up care in orthopedic and trauma surgery with video consultations: health economic evaluation study from a health provider’s perspective. J Med Internet Res.

